# Diastolic dysfunction precedes hypoxia-induced mortality in dystrophic mice

**DOI:** 10.14814/phy2.12513

**Published:** 2015-08-26

**Authors:** DeWayne Townsend

**Affiliations:** Department of Integrative Biology and Physiology, University of Minnesota Medical SchoolMinneapolis, Minnesota

**Keywords:** Duchenne muscular dystrophy, dystrophic cardiomyopathy, dystrophin, hypoxia, right ventricle

## Abstract

Duchenne muscular dystrophy (DMD) is a progressive striated muscle disease that is characterized by skeletal muscle weakness with progressive respiratory and cardiac failure. Together respiratory and cardiac disease account for the majority of mortality in the DMD patient population. However, little is known regarding the effects of respiratory dysfunction on the dystrophic heart. The studies described here examine the effects of acute hypoxia on cardiac function. These studies demonstrate, for the first time, that a mouse model of DMD displays significant mortality following acute exposure to hypoxia. This mortality is characterized by a steady decline in systolic function. Retrospective analysis reveals that significant decreases in diastolic dysfunction, especially in the right ventricle, precede the decline in systolic pressure. The initial hemodynamic response to acute hypoxia in the mouse is similar to that observed in larger species, with significant increases in right ventricular afterload and decreases in left ventricular preload being observed. Significant increases in heart rate and contractility suggest hypoxia-induced activation of the sympathetic nervous system. These studies provide evidence that while hypoxia presents significant hemodynamic challenges to the dystrophic right ventricle, global cardiac dysfunction precedes hypoxia-induced mortality in the dystrophic heart. These findings are clinically relevant as the respiratory insufficiency evident in patients with DMD results in significant bouts of hypoxia. The results of these studies indicate that hypoxia may contribute to the acceleration of the heart disease in DMD patients. Importantly, hypoxia can be avoided through the use of ventilatory support.

## Introduction

Duchenne muscular dystrophy (DMD) is a progressive disease of striated muscle deterioration. Initially presenting as skeletal muscle weakness, the disease advances resulting in the loss of ambulation early in the second decade of life and death in the third or fourth decade (Bushby et al. 2003; Eagle et al. 2007). Respiratory failure has been the leading cause of mortality in DMD since its first description in the nineteenth century (Duchenne 1867; Clarke and Gowers 1874; McCormack and Spalter 1966; Inkley et al. 1974). However, recent advances in symptomatic respiratory therapy have resulted in significant extension of life for DMD patients (Jeppesen et al. 2003; Eagle et al. 2007). With this prolonged life-span, the concurrent development of cardiac dysfunction has become more apparent. Cardiac disease was also noted in many early descriptions of DMD patients (Ross 1883; Globus 1923), but understanding of the pathophysiology of cardiac disease has lagged behind that of skeletal muscle. The natural history of the disease is such that respiratory and cardiac dysfunction develop in parallel, both becoming clinically evident sometime after the loss of ambulation. Surveys of providers reveal that over 70% of DMD patients display symptoms of respiratory disease before referral for respiratory therapy (Finder et al. 2004; Bersanini et al. 2012; Katz et al. 2013) and even in patients with normal daytime pulmonary function tests, nocturnal hypoxia can occur to a significant degree (Katz et al. 2010; Bersanini et al. 2012). Thus, even with good medical management, DMD patients routinely have bouts of hypoxia (Bushby et al. 2003, 2004, 2010a, 2010b). The respiratory failure seen in DMD patients results from hypoventilation of the alveolus secondary to weakened respiratory muscles. This results in a build up of CO_2_ and a reduction of O_2_ in the blood. The increased CO_2_ results in a respiratory acidosis, which is partially compensated for by the kidneys. However, there is no alternative source of O_2_, thus the hypoxia present in dystrophic respiratory failure is particularly important.

The mdx mouse is a genetic model of DMD that displays myopathic changes and reduced skeletal muscle specific force generation (Bulfield et al. 1984; Lynch et al. 2001). Furthermore, these mice have significant reductions in cardiac function (Lu and Hoey 2000; Quinlan et al. 2004; Meyers and Townsend 2015) and significant reductions in respiratory function (Farkas et al. 2007; Ishizaki et al. 2008; Huang et al. 2011). Previous work has demonstrated that mild hypoxia results in significant dysfunction (Farkas et al. 2007) and apoptosis (Kozłowska et al. 1999) in the diaphragm, but the effect of hypoxia on the dystrophic heart has not been investigated. In the studies presented here we use the mdx mouse to assess the pathophysiological importance of hypoxia. The most direct link between cardiac function and hypoxia is mediated through the constriction of the pulmonary vasculature during hypoxic exposure (Bergofsky et al. 1963). Increases in pulmonary vascular resistance will increase the afterload upon the right ventricle, increasing the pressure required to maintain a constant cardiac output. The left ventricle of the dystrophic heart is particularly susceptible to injury following increases in afterload generated by abdominal aortic constriction (Kamogawa et al. 2001). These data suggest that increased strain on the right ventricle caused by hypoxic vasoconstriction of the pulmonary vasculature may cause similar damage in the right ventricle.

Hypoxia is an important component of the respiratory insufficiency that is extremely prevalent in the advanced stages of DMD and understanding the cardiac consequences of this hypoxia on the dystrophic heart is of particular importance. In the current studies, it is demonstrated that the dystrophic right ventricle initially displays a normal hemodynamic response to acute hypoxia. However, after a short period of hypoxia, dystrophic mice display a significant level of mortality compared to wild type mice. These observations have immediate clinical relevance as they suggest that the short bouts of hypoxia experienced by DMD patients may contribute to myocardial dysfunction and damage. These data implicate hypoxia as a potential inciting factor in the development of dystrophic cardiomyopathy and suggests that earlier initiation of respiratory support could delay the onset of heart disease in DMD patients.

## Material and Methods

### Animals

Control (C57BL/10) and mdx (C57BL/10 ScSn-Dmd^mdx^/J) mouse colonies were established from breeding pairs purchased from Jackson Laboratories (Bar Harbor, ME) and maintained at the University of Minnesota. Genetic makeup of these colonies was maintained by replacing the breeding stock every four generations. Animals were fed standard chow diet ad libitum and were kept in a room with 12-h light and dark cycles. These studies used mice of both sexes aged 4–6 months. All procedures reported here were reviewed and approved by the University of Minnesota Institutional Animal Care and Use Committee.

### In vivo hypoxia: hemodynamics

To assess the hemodynamic effects of hypoxia in the dystrophic mouse, biventricular cardiac catheterization was performed under normoxic and hypoxic conditions. Mice were instrumented as described previously (Meyers and Townsend 2015). Briefly, mice were intubated and ventilated by a positive pressure ventilator with 4 cm H_2_O PEEP (Kent Scientific, Torrington, CT). This allowed tight control of the content of respiratory gases being inhaled. A 1.2-Fr pressure-volume catheter (Scisense, Ithaca, NY) was inserted into the left ventricle and 1.2-Fr pressure catheter (Scisense, Ithaca, NY) was inserted into the right ventricle, both through apical stab incisions. Mice were fitted with a pulse oximeter located on their right thigh (Starr Life Sciences, Holliston, MA). Following instrumentation, the isoflurane was set to 1%, a level sufficient to maintain deep sedation. Overall surgical time from induction to the beginning of the protocol was about 45 min. Gas mixtures were generated using a custom built device consisting of two flow meters, one controlling oxygen and the other controlling nitrogen. The gas mixtures were calibrated using a ProOx P110 oxygen sensor (Biospherix, Lacona, NY) with a correlation coefficient of 0.996. Respiratory rate was set to be the lowest rate that prevented respiratory effort in the anesthetized mice. Mortality was defined as a left ventricular systolic pressure of <40 mmHg.

### Statistical analysis

All data were analyzed using either R (R Core Team 2015) or Prism 5 software (GraphPad Software, Inc., La Jolla, CA). Where multiple comparisons were performed, one-way ANOVA followed by a Bonferroni post test was used. Comparisons between two groups used Student’s *t*-test. Kaplan**–**Meier method was used to create survival curves, followed by a Log-rank (Mantel-Cox) test to determine differences in survival. The box and whisker plots in several of the figures show the distribution of the data. In these plots, the box defines the 25–75 percentile spread, the line in the middle is the median, and the “+” is the mean. The lines extent out to the maximum and minimum values, however, points outside of 1.5 the interquartile distance are displayed separately, but are included in any relevant statistical comparisons.

## Results

Recently published work demonstrates the increased level of fibrosis within the right ventricle of the mdx mouse (Meyers and Townsend 2015). It is hypothesized that this increased damage to the dystrophic right ventricle is occurring secondary to hypoxic pulmonary arterial constriction which increases the afterload on the right heart and that this results in an increased level of myocardial damage. The current study assesses the hemodynamic consequences of acute hypoxia in both wild type and dystrophic hearts. Following instrumentation and an equilibration period, hemodynamic data were collected with a fraction of inhaled oxygen (FIO_2_) of 100%. The FIO_2_ was decreased to 20% for 10 min and then finally to 10%. Detailed hemodynamic analysis was performed following 10 min at an FIO_2_ of 10%. This level of hypoxia results in a significant desaturation of hemoglobin in both wild type (98.1 ± 0.3% to 66.7 ± 4.5%) and dystrophic mice (97.7 ± 0.2% to 64.0 ± 3.4%). Importantly, there is no significant difference in the degree of hemoglobin desaturation observed between wild type and dystrophic mice, indicating an equivalent level of hypoxia.

**Figure 1:**
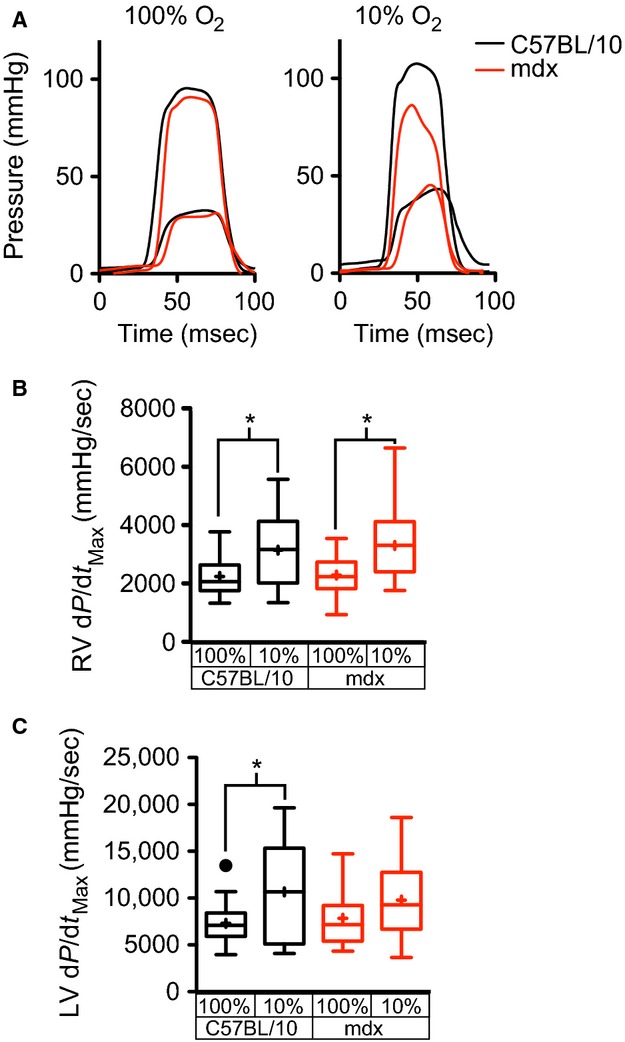
Effects of acute hypoxia on the systolic function of both right and left ventricles of wild type and dystrophic mice. Sample waveforms from both right and left ventricles of wild type (black) and dystrophic (red) hearts with 100% O_2_ (A). 10 min following the introduction of hypoxia, the maximum d*P*/d*t* of the right ventricle of both wild type and dystrophic hearts are significantly elevated (B). The left ventricles of wild type hearts, but not dystrophic hearts, demonstrate a significant increase in maximum dP/dt with acute hypoxia (C). These studies examined the hypoxic response of 27 mdx and 23 C57BL/10 mice. *P < 0.05 by two-way ANOVA with post-hoc testing.

Assessment of biventricular pressure allows for a detailed analysis of the hemodynamic consequences of acute hypoxia. Under normoxic conditions, there are no significant differences in the dP/dt_Max_ of either ventricle in wild type and dystrophic mice (Fig.1B and C). Introduction of hypoxia results in an elevation of right ventricular (RV) d*P*/d*t*_Max_ (Fig.1B) in both wild type and dystrophic hearts, consistent with an increase in afterload secondary to hypoxia-induced pulmonary arterial constriction. In wild type hearts, but not dystrophic hearts, hypoxia results in a significant increase in LV d*P*/d*t*_Max_ (Fig.1C), this lack of inotropic response in the dystrophic hearts likely results from a reduced cardiac reserve in these dystrophic mice. Importantly, hypoxia has no significant effects on RV end-diastolic pressures (EDP) in wild type (4.85 ± 0.43 mmHg vs. 5.48 ± 0.59 mmHg for normoxic and hypoxic conditions, respectively) or mdx (3.48 ± 0.55 mmHg vs. 4.20 ± 0.70 mmHg for normoxic and hypoxic conditions, respectively) hearts.

**Figure 2:**
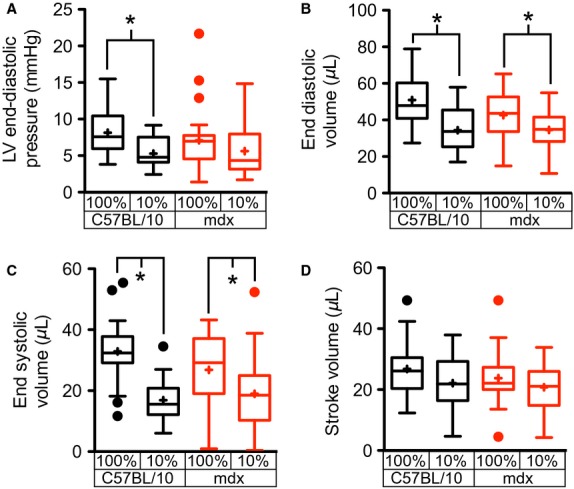
Reductions in left ventricular loading following induction of acute hypoxia. Measures of left ventricular loading following the induction of acute hypoxia demonstrate a significant decline of left ventricular end-diastolic pressure in wild type hearts (A). Both wild type and dystrophic left ventricles display significant reductions in end-diastolic volume following initiation of hypoxia (B). This change in end-diastolic volume is offset by a similar decrease in end-systolic volume (C) resulting in a maintenance of the ventricular stroke volume (D). These studies examined the hypoxic response of 27 mdx and 23 C57BL/10 mice. *P < 0.05 for the indicated comparison.

The hypoxia-induced increase in RV afterload results in changes in the loading of the LV. In wild type hearts, hypoxia results in a significant decline in LV end-diastolic pressure (EDP; Fig.2A) and minimum pressure (3.44 ± 0.34 vs. 2.30 ± 0.39 for normoxic and hypoxic conditions, respectively). In dystrophic hearts, acute hypoxia has no significant effects on the LV-EDP. Although 73% of dystrophic mice demonstrated a decline in LV-EDP with hypoxia, variability in the magnitude of this decline and several mice where hypoxia increased LV-EDP prevent statistically significant differences in the dystrophic mice as a whole. This decline in left ventricular preload is further evidenced by significant reductions in left ventricular end-diastolic volume (LV-EDV) in both wild type and dystrophic mice (Fig.2B). The significant reduction in LV-EDV between wild type and dystrophic hearts is observed under normoxic conditions (Yasuda et al. 2005; Townsend et al. 2007; Crisp et al. 2011) is completely lost with the introduction of acute hypoxia. This decline in the LV-EDV is accompanied by a significant reduction in the left ventricular end-systolic volume (LV-ESV) in both wild type and dystrophic hearts (Fig.2C). These changes in LV volume result in a small nonsignificant decrease in the stroke volume (Fig.2D).

**Figure 3:**
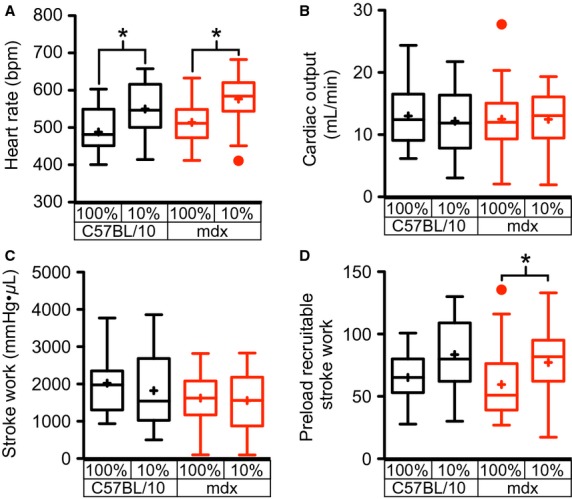
Elevations in heart rate and contractility with acute hypoxia. Ten minutes following the initiation of acute hypoxia, a significant elevation in heart rate is observed in both wild type and dystrophic hearts (A). No significant differences in cardiac output (B) or stroke work (C) were observed in either genotype. In dystrophic hearts an increase in the preload recruitable stroke work, a load independent measure of contractility, was detected (D). These results are consistent with an increased sympathetic nervous system activation coupled to a decline in left ventricular preload. These studies examined the hypoxic response of 27 mdx and 23 C57BL/10 mice. *P < 0.05 for the indicated comparisons.

**Figure 4:**
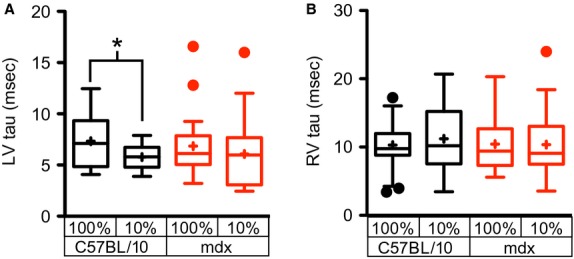
Effects of acute hypoxia on diastolic function of the right and left ventricle. Ten minutes following acute hypoxia, there is a significant decrease in the relaxation time constant in wild type hearts, but not in the dystrophic left ventricle (A). In the right ventricle, there is no change in the exponential decay time constant tau with hypoxia in the right ventricle of either wild type or dystrophic hearts (B). These studies examined the hypoxic response of 27 mdx and 23 C57BL/10 mice. *P < 0.05 for the indicated comparisons.

The hypoxia-induced reductions in LV volumes, coupled with the maintenance of systolic function, suggest that the sympathetic nervous system becomes activated and increases the contractile function of the heart in response to hypoxia. The significant increase in heart rate observed in both wild type and dystrophic hearts in response to hypoxia (Fig.3A), is consistent with this model. The increase in heart rate and small decline in stroke volume (Fig.2D) leave cardiac output essentially unchanged (Fig.3B). Consistent with the maintenance of systolic pressures and stroke volume, hypoxia has no significant effect on the left ventricular stroke work (Fig.3C). The load independent measure of contractility, preload-recruitable stroke work, is significantly increased during acute hypoxia in dystrophic hearts and trending upward in wild type hearts (Fig.3D). These data are consistent with an increased activation of the cardiac sympathetic nervous system during hypoxia. In wild type hearts, LV relaxation, as measured by Tau, is accelerated by hypoxia (Fig.4A), but this lusitropic response is absent in the dystrophic heart. Interestingly, hypoxia has no significant effect on the relaxation of the RV in either wild type or dystrophic hearts (Fig.4B).

**Figure 5:**
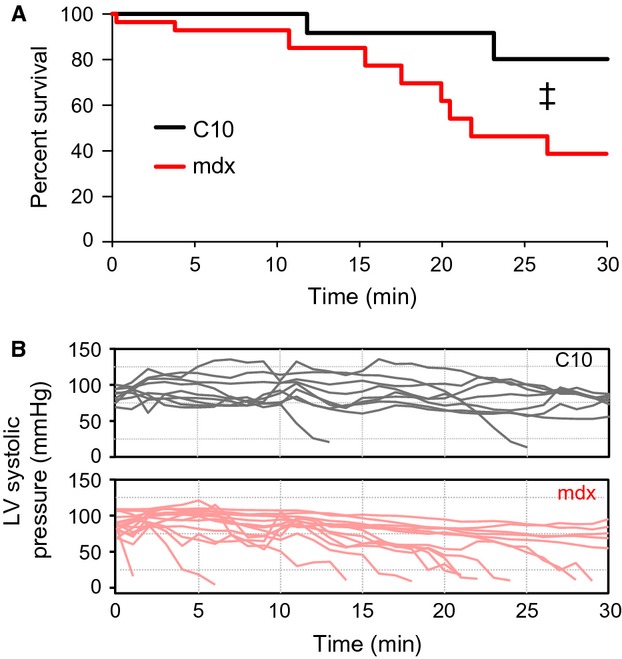
Significant increased hypoxia-induced mortality in dystrophic mice. With prolonged hypoxic exposure dystrophic mice display significantly greater level of mortality compared to wild type mice (A). Select representative tracings of left ventricular systolic pressure (B), over time during the hypoxia challenge. This analysis used survival data from 28 mdx and 24 C57BL/10 mice. In both plots, hypoxia begins at time zero. ^‡^*P* = 0.036 via the log-rank test.

**Figure 6:**
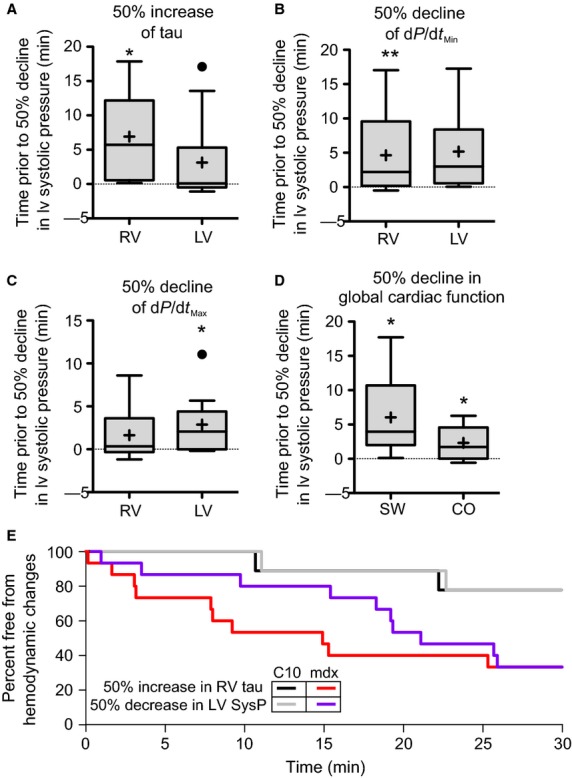
Retrospective analysis of dystrophic mice that displayed hypoxia-induced mortality. Examining hemodynamics of only the dystrophic mice that died during the hypoxic challenge reveals parameters that were leading indicators of impending cardiac decompensation. For these analysis, the time for a hemodynamic parameter to increase or decrease by 50% relative to baseline levels was compared to the time to 50% reduction of left ventricular systolic pressure. These analysis indicated that declines in diastolic function significantly proceeded the loss of LV systolic pressure in both ventricles (A and B). The maximal rate of pressure development (d*P*/d*t*_Max_) in the left ventricle, but not the right ventricle, decreases significantly prior to declines in left ventricular systolic pressure (C). Declines in global cardiac function also significantly precede the decline in LV systolic pressure (D). *P < 0.05 that the time preceding loss of 50% LV systolic pressure significantly different from zero. (E) Analysis of survival curves using either the time to 50% increase in RV Tau or 50% decrease in LV end-systolic pressure applied to all mice in the survival study.

Extension of the hypoxic period induces a significant mortality in dystrophic mice (Fig.5A). This mortality is characterized by a gradual decline in left ventricular end-systolic pressure (Fig.5B). Further analysis of the end-stage hemodynamics reveal evidence of eventual RV failure characterized by increases in RV-EDP (8.32 ± 0.12 in the decompensated heart vs. 4.67 ± 0.20 at baseline). However, at this late stage there is clear evidence of systolic dysfunction in both right and left ventricles with significant declines in systolic pressures and d*P*/d*t*_Max_. Importantly, there is a significant reduction in LV-EDP and LV-EDV, consistent with a decline in LV preload resulting from RV dysfunction. In order to begin to understand the physiological processes involved in decompensation of the dystrophic heart during hypoxia, the hemodynamic data leading up to decompensation were analyzed in a retrospective fashion. The time at which each parameter increased or decreased by 50% of the prehypoxic levels were compared to the time at which LV-ESP declined below 50% of the prehypoxic LV-ESP. Several hemodynamic parameters were identified that significantly preceded the decline of LV systolic pressure. Most notable of these is a 50% increase in RV tau that preceded the decline in LV-ESP by 6.9 ± 2.1 min (Fig.6A). Interestingly, changes in LV tau do not significantly precede the decline in LV-ESP. However, declines in d*P*/d*t*_Min_ in both ventricles suggest that diastolic dysfunction is an early indicator of impending decompensation in both the RV and LV (Fig.6B). There are several sensitive indicators of LV systolic function that precede reductions in LV-ESP, specifically reductions in d*P*/d*t*_Max_, cardiac output, and stroke work all decline significantly earlier than LV-ESP (Fig.6C and D). To assess the specificity of the decline in RV diastolic function to predict decompensation, the survival analysis was recalculated using the time to a 50% decline in LV-ESP and a 50% increase in RV-Tau. In dystrophic mice, the use of increases in RV-Tau results in a leftward shift in the survival curve. Importantly, all mice that experienced a 50% increase in RV-Tau eventually decompensated. Interestingly, in the few wild type mice that decompensated during the hypoxic protocol, increases in RV-Tau did not precede declines in LV-ESP. This suggests that the increase RV-Tau is specific to dystrophic mice and is not a feature of decompensating hearts.

## Discussion

The central observation of these studies is the first description of hypoxia-induced mortality in the dystrophic mouse. It is demonstrated that in young mdx mice reductions in oxygen are sufficient to result in cardiac decompensation. Dystrophic mice of the age used in this study are largely free of structural cardiac disease (Quinlan et al. 2004; Meyers and Townsend 2015), so this rapid decline is surprising. Furthermore, this finding is particularly important given the high prevalence of hypoxia in the DMD patient population (Bushby et al. 2003, 2004, 2010a, 2010b). Cardiomyopathy is a leading cause of mortality in patients with DMD, thus understanding the basic mechanisms underlying the progression of heart disease in these patients is of critical importance. Respiratory insufficiency is a significant complication of DMD and presents unique hemodynamic and metabolic challenges for the heart. This study demonstrates that the dystrophic process increases the susceptibility of the heart to hypoxia.

Using a novel biventricular catheterization preparation during the hypoxic challenge provides a very detailed picture of the hemodynamic response to acute hypoxia. It is demonstrated that the initial hemodynamic response to acute hypoxia is relatively equal between wild type and dystrophic hearts. With both wild type and dystrophic hearts demonstrating increases in right ventricular afterload and decreases in left ventricular preload. Despite this relatively normal initial hemodynamic response, dystrophic mice are unable to sustain normal contractile function during prolonged hypoxic exposure. The actual cause of death is an interesting question. Assessing the dystrophic mice in the late stages of decompensation it is clear that they are in acute heart failure, characterized by poor left ventricular systolic function and increases in right ventricular diastolic pressure. The processes resulting in this state of heart failure is less well-defined.

Retrospective analysis of hemodynamic data from dystrophic mice that decompensated, there is evidence of biventricular diastolic dysfunction occurring 5–7 min prior to the decline in left ventricular systolic pressure. This diastolic dysfunction is evident slightly earlier in the RV, raising the possibility that the RV dysfunction may be an initiating cause of the global cardiac failure. Failure of the right heart would be expected to result in a decrease in the preload filling the left ventricle. However, declines in measures of left ventricular preload (LV-EDP and LV-EDV) decline in parallel with LV systolic pressure. Furthermore, the measures of global cardiac function, stroke work and cardiac output, both begin to decline several minutes prior to the loss of LV systolic pressure or alterations of LV preload. Together these data support a model where hypoxia-induced cardiac decompensation begins with declines in diastolic function, initially in the right ventricle, perhaps secondary to the additional hemodynamic demands placed on this ventricle during hypoxia. However, this diastolic dysfunction is also observed in the left ventricle a few minutes later. Following these changes in diastolic function, several measures of LV systolic function begin to decline, in the absence of changes in LV preload. The observation that hypoxia results in diastolic dysfunction prior to declines in systolic function in both ventricles of the dystrophic heart, suggests that the hypoxia-induced hemodynamic changes in pulmonary vascular resistance do not contribute significantly to the cardiac decompensation observed in the hypoxic dystrophic heart. Hypoxia-induced declines in left ventricular preload would be expected to decrease systolic function initially. These data support a model whereby hypoxia induces biventricular reductions in cardiac function, initially presenting as diastolic dysfunction and followed by declines in contractile function.

The nature of the mechanism of cardiac decompensation and death in mdx mice exposed to hypoxia is not clear. The data presented here provide a detailed hemodynamic picture leading up to the decline in left ventricular systolic function. This study and others (Nahas et al. 1954; Marshall and Metcalfe 1988; Hanada et al. 2003; Pearson et al. 2007) demonstrate that there is a significant increase in heart rate in response to hypoxia secondary to sympathetic activation. Furthermore, this hypoxia-induced elevation in sympathetic drive is transient in nature, lasting just a few minutes (Marshall and Metcalfe 1988; Campen et al. 2005). There is significant evidence indicating that excessive sympathetic stimulation could contribute to cardiac damage in the dystrophic heart (Danialou et al. 2001; Yasuda et al. 2005; Townsend et al. 2007; Strakova et al. 2014). Dystrophic mice also have an increased basal sympathetic tone (Chu et al. 2002) so it is also possible that the withdrawal of sympathetic activity after a few minutes of hypoxia may also contribute to the eventual cardiac decompensation in the dystrophic mouse heart.

The use of biventricular measurements provides unique insight into the interaction between the right and left ventricles of the dystrophic mouse heart. It has been demonstrated that the left ventricular end-diastolic volume (LV-EDV) of young dystrophic mice is significantly decreased relative to wild type mice (Yasuda et al. 2005; Townsend et al. 2007; Crisp et al. 2011). The initial interpretation of this observation was a decrease in compliance of the left ventricular wall. However, subsequent studies have demonstrated that the intact dystrophic heart has significantly increased passive compliance (Barnabei and Metzger 2012; Meyers and Townsend 2015). The current study reveals that the pressures developed by the RV under baseline conditions are equivalent to that of wild type mice. These data suggest that differences in the pulmonary circulatory resistance may contribute to the decrease in preload in the dystrophic heart under baseline conditions. During the current studies both wild type and dystrophic mice were ventilated and hemoglobin saturations confirm equivalent oxygenation, indicating sufficient delivery of oxygen to the alveoli during the course of the study. However, it is well-documented that the respiratory muscles of the mdx mouse display a very severe pathology and that this pathology is associated with reductions in respiratory function (Ishizaki et al. 2008; Mosqueira et al. 2013). Chronic exposure to hypoxia, such as that which occurs with hypoventilation, causes long-term remodeling of the pulmonary vasculature resulting in a persistent narrowing of the pulmonary arteries (Naeije 2010; Cahill et al. 2012). It is possible that chronic remodeling of pulmonary arterial tone by either hypoxia or a direct effect of dystrophin’s absence in the pulmonary vasculature could contribute to the decline in preload to the left ventricle in mdx mice, yet be mild enough to not generate significant levels of pulmonary hypertension. This model is supported by evidence that right ventricular end-systolic volume is significantly elevated in mdx mice (Crisp et al. 2011), consistent with an increase in right ventricular afterload. Interestingly, this increase RV end-systolic volume is normalized in mdx mice expressing utrophin in their skeletal muscle (Crisp et al. 2011), suggesting that the improved ventilation resulting from the correction of pathology may decrease RV afterload. Dehydration or a loss of blood volume could also result in a relative decrease in ventricular volumes. However, there is no evidence of a reduction in the right ventricular preload that would be expected from a global reduction in blood volume.

In summary, the studies described here identify a novel hypoxia sensitivity of the dystrophic heart that affects both ventricles, suggesting some aspect of global cardiac dysfunction. Further studies are required to better understand the underlying mechanism by which hypoxia results in declines in cardiac function and eventual mortality. However, the observed hypoxia-induced cardiac decompensation observed in the mdx mouse raises important concerns regarding the role of hypoxia in the progression of dystrophic cardiomyopathy in DMD patients.

## Conflict of Interest

None declared.
